# Randomized and dose-escalation trials of recombinant human serum albumin /granulocyte colony-stimulating factor in patients with breast cancer receiving anthracycline-containing chemotherapy

**DOI:** 10.1186/s12885-021-08093-z

**Published:** 2021-03-31

**Authors:** Shanshan Chen, Yiqun Han, Quchang Ouyang, Jianguo Lu, Qingyuan Zhang, Shun’e Yang, Jingfen Wang, Haixin Huang, Hong Liu, Zhimin Shao, Hui Li, Zhendong Chen, Sanyuan Sun, Cuizhi Geng, Junguo Lu, Jianwei Sun, Jiayu Wang, Binghe Xu

**Affiliations:** 1 Department of Medical Oncology, National Cancer Center/ National Clinical Research Center for Cancer/ Cancer Hospital, Chinese Academy of Medical Sciences and Peking Union Medical College, No. 17 Panjiayuan Nanli, Chaoyang District, Beijing, 100021 China; 2 Department of Breast Cancer Medical Oncology, Hunan Cancer Hospital, Changsha, Hunan China; 3 Department of General Surgery, Xi’an Tangdu Hospital, Xi’an, Shanxi China; 4 Department of Oncology, Harbin Medical University Cancer Hospital, Harbin, Heilongjiang China; 5 Department of Breast Cancer and Lymphoma, The Affiliated Tumor Hospital of Xinjiang Medical University, Urumqi, Xinjiang China; 6 Department of Internal Medicine, Linyi Tumor Hospital, Linyi, Shandong China; 7 Department of Oncology, The Fourth Affiliated Hospital of Guangxi Medical University, Liuzhou, Guangxi China; 8 Department of Breast Surgery, National Clinical Research Center for Cancer, Tianjin Medical University Cancer Institute and Hospital, Tianjin, China; 9 Department of Breast Surgery, Fudan University Shanghai Cancer Center, Shanghai, China; 10 Department of Breast Surgery, Sichuan Province Tumor Hospital, Chengdu, Sichuan China; 11 Department of Medical Oncology, The Second Affiliated Hospital of Anhui Medical University, Hefei, Anhui China; 12 Department of Medical Oncology, Central Hospital of Xuzhou, the Cancer Institute of Southeast University, Xuzhou, Jiangsu China; 13 First Department of Surgery, The Fourth Hospital of Hebei Medical University, Shijiazhuang, Hebei China; 14 Department of Medical Oncology, Nantong Tumor Hospital, Nantong, Jiangsu China; 15 Department of Tumor, Yunnan First People’s Hospital, Kunming, Yunnan China

**Keywords:** Breast cancer, Chemotherapy, Clinical trials, Neutropenia, rHSA/G-CSF, rhG-CSF

## Abstract

**Background:**

To evaluate the efficacy and safety of recombinant human serum albumin /granulocyte colony-stimulating factor (rHSA/G-CSF) in breast cancer following receipt of cytotoxic agents.

**Methods:**

The phase 1b trial assessed the pharmacokinetics, pharmacodynamics, and safety of dose-escalation, ranging from rHSA/G-CSF 1800 μg, 2100 μg, and 2400 μg. Randomized controlled phase 2b trial was further conducted to ensure the comparative efficacy and safety of rHSA/G-CSF 2400 μg and rhG-CSF 5 μg/kg. In multicenter, randomized, open-label, parallel, phase 2 study, participants treated with anthracycline-containing chemotherapy were assigned in a ratio 1:1:1 to receive double delivery of rHSA/G-CSF 1200 μg, 1500 μg, and continuous rhG-CSF 5 μg/kg.

**Results:**

Between December 16, 2014, to July 23, 2018, a total of 320 patients were enrolled, including 25 individuals in phase 1b trial, 80 patients in phase 2b trial, and 215 participants in phase 2 study. The mean duration of agranulocytosis during the first chemotherapeutic intermission was observed as 1.14 ± 1.35 days in rHSA/G-CSF 1500 μg, which was comparable with that of 1.07 ± 0.97 days obtained in rhG-CSF control (*P* = 0.71). Safety profiles were assessed to be acceptable ranging from rHSA/G-CSF 1800 μg to 2400 μg, while the double delivery of HSA/G-CSF 2400 μg failed to meet the noninferiority in comparison with rhG-CSF.

**Conclusion:**

The prospective randomized controlled trials demonstrated that rHSA/G-CSF was efficacious and well-tolerated with an approachable frequency and expense of application for prophylactic management of agranulocytosis. The double delivery of rHSA/G-CSF 1500 μg in comparisons with paralleling G-CSF preparations is warranted in the phase 3 trial.

**Trial registration:**

ClinicalTrials.gov identifiers: NCT02465801 (11/17/2014), NCT03246009 (08/08/2017), NCT03251768  (08/07/2017).

**Supplementary Information:**

The online version contains supplementary material available at 10.1186/s12885-021-08093-z.

## Background

Over the past decades, the application of cytotoxic agents has provided great benefits for almost all categories of malignancies in clinical practice [1, 2]. However, this objective efficacy could be dispensed a reduction as a result of drug-related toxicities, of which neutropenia is a crucial proportion. Although neutropenia tends to be asymptomatic, serious clinical sequelae could be ascribed to it, including febrile neutropenia (FN), sepsis, an increased risk of opportunistic infection, which could be serious and beyond management [3].

Recombinant granulocyte-colony stimulating factor (G-CSF) preparations have emerged as effective supportive therapies for chemotherapy-induced neutropenia, of which the clinical efficacy has been confirmed by numerous clinical trials, and generally recommended by guidelines [4–9]. Through binding to the specific transmembrane receptor, G-SCF exerts its role by modulating the proliferation and differentiation of committed progenitor cells to granulocytes and functionally stimulating mature neutrophils [10, 11]. The biosimilar recombinant human G-CSF (rhG-CSF), possessing a similar biological mechanism, has been successfully approved for application in reducing the risk and duration of chemotherapy-induced neutropenia and febrile neutropenia [12, 13]. Because of the receptor-mediated elimination, kidney clearance, and enzymatic degradation mechanism, the half-life of rhG-CSF is shortened (4 to 8 h) that patients had to object frequent subcutaneous injections to maintain effective concentration [14, 15]. Accordingly, novel strategies for durative effectiveness are anticipated for clinical practice.

Recently, the pegylated G-CSF, as a covalent conjugate of rhG-CSF and polyethylene glycol (PEG), has been designed and developed to improve the systemic exposure to the drug delivery and further enhance the biological response to effective components. This novel structure of pegylation could weaken the proteases hydrolysis toward protein components and invariably prolong the half-life of the agent (~ 72 h) [16–18]. However, this merit could be partially offset by the steric hindrance effect of PEG, leading to a decrease in bioactivity and attribution of drug-related toxicities [19]. By virtue of a durative half-life over approximately 19 days in vivo, human serum albumin (HSA) has become a novel source of fusion partners [20, 21]. This enduring parameter tends to lie in the mechanisms of the close interaction between HSA and the neonatal Fc receptor, which further protects HSA against lysosome degradation [22]. The increased molecular weight of the compound and mask of HSA have allowed for long-lasting agents, including the fusion of serum albumin with glucagon-like peptide-1 (Albiglutide) and the albumin-bound paclitaxel (Abraxane) [23–25].

Recombinant human serum albumin /granulocyte colony-stimulating factor (rHSA/G-CSF) was a novel biosimilar product of rhG-CSF, which was developed on the basis of a proprietary recombinant DNA process that was expressed in highly engineered yeast strain *Pichia pastoris.* Preclinical studies have suggested that the elimination half-life of rHSA/G-CSF (~ 38.6 h) is approximately over 10 times longer than that of rhG-CSF (~ 2.54 h). The double dose of rHSA/G-CSF administered at the interval of 96 h has comparable efficacy on the motivation of circulating neutrophils with standard rhG-CSF continuously administered once a day.

Herein, we conducted a study with three proportions of phase 1b trial, phase 2b trial, and phase 2 trial, to evaluate the safety, pharmacokinetics (PK), efficacy, and pharmacodynamics (PD) of rHSA/G-CSF in breast cancer patients receiving anthracycline-containing chemotherapy.

## Methods

### Study overview

This three-stage study comprised a dose-escalation study (phase 1b), a dose-expansion study (phase 2b), and a randomized controlled study (phase 2). A dose-escalation study was performed to assess the dose-dependent effect, observed in the aforementioned trial, and explored the safety, PK, PD of the growing dosage rHSA/G-CSF. Phase 2b trial involved an expanding cohort was to assess the comparative efficacy and safety of the regimens containing rHSA/G-CSF, based on the recommended dosage according to prior assessment, and the paralleling rhG-CSF. Patients enrolled in phase 2 trial were treated with successive increasing doses of rHSA/G-CSF or the paralleling controlled agent (rhG-CSF), of which the findings recommended the non-inferior dosage regimens of rHSA/G-CSF in terms of prevention from agranulocytosis following anthracycline-containing chemotherapy.

This study was conducted in consistence with protocol, good clinical practice, and the Declaration of Helsinki. The protocol and corresponding amendments were approved by individualized review boards or ethics committees of participating institutions. All participants have provided written informed consent prior to enrollment. The experimental agent-rHSA/G-CSF-was provided by Tianjin SinoBiotech Ltd., a subsidiary of FortuneRock (China) Co., Ltd. The paralleling-controlled drug (Jisaixin) was provided by North China Pharmaceutical Jintan Biotechnology Co., Ltd. with 300 μg/tube.

### Study population and design

Eligible patients were aged 18 to 65 years with histologically confirmed stage I-III after breast conservative or radical surgery or de novo stage IV breast cancer; treatment-naïve and scheduled to receive chemotherapy; an absolute neutrophil count (ANC) ≥ 1.5 × 10^9^/L, platelet ≥100 × 10^9^/L at baseline; East Cooperative Oncology Group (ECOG) performance status of 0 or 1, and no bone marrow involvement. Patients were excluded if they received radiotherapy (excluding local radiotherapy for bone metastasis) within 4 weeks before enrollment; suffering the infectious disease in uncontrollability, and with axillary temperature ≥ 38 °C; durative diabetes mellitus, with the fasting serum level of glycose beyond the normal range; presenting the history of allergic to protein pharmaceutical preparations, and human chorionic gonadotropin testing positive in fertile women.

Participants were enrolled in the phase 1b study (ClinicalTrials.gov identifier: NCT03246009) between May 23, 2016, to April 10, 2017, constituting two cohorts, in which rHSA/G-CSF was administrated on the single and double dosage in sequential dose-escalation design, and both the dosage regimens were on the basis of rHSA/G-CSF 1800 μg, 2100 μg, and 2400 μg to assess the dose-dependent effect and explore the safety, efficacy, PK, and PD. Based on the ‘3 + 3’ principle, eligible patients with leukopenia or neutropenia following anthracycline-containing chemotherapy were successively allocated initiated at the 1800 μg with subsequent doses studied in an ascending manner at an interval of 10 days. At a follow-up of 14 days, patients were observed for clinical and laboratory manifestations. The multi-dose study was based on the results of the single-dose study, in which patients in two dose cohorts were scheduled to receive repeatedly given doses of rHSA/G-CSF at an interval of 4 days until a presentation of white blood cell (WBC) ≥ 10 × 10^9^/L or ANC ≥ 5 × 10^9^/L. The dosage was consecutively escalating if none of the patients at a given dose experienced dose-limiting toxicity (DLT). If two or more patients experienced a DLT, dose-escalation would cease for further enrollment at that dosage level. Any cohort in which one patient experienced DLT was expanded by up to six patients. If any one of the six patients exhibited evidence of a DLT, dose escalation was stopped and no further treatments were administered. Patients were considered as treatment failure if they presented with a grade 3–4 leukopenia or neutropenia on the 4th day following administration of rHSA/G-CSF. These patients would be withdrawn from the study and received standard supportive care of rhG-CSF, and the continuation or a failed termination of the study, for the patients allocated in the post dose, would be determined by investigators.

The phase 2b trial was a multicenter, randomized, open-label, parallel study (ClinicalTrials.gov identifier: NCT03251768), of which breast cancer patients suitable for chemotherapy based on TEC or TE regimens were recruited between October 19, 2017, to July 23, 2018, and randomly allocated in a 1:1 ratio to two groups that were treated with either rHSA/G-CSF 2400 μg or rhG-CSF (Jisaixin) 5 μg/kg in the same pattern of administration on the chemotherapeutic intermissions.

The phase 2 trial was a multicenter, randomized, open-label, paralleling-controlled study with the prospective registration on ClinicalTrials.gov (ClinicalTrials.gov identifier: NCT02465801). Eligible patients were enrolled between December 16, 2014, to December 23, 2015, and randomly assigned in a ratio of 1:1:1 to prophylactically receive twice rHSA/G-CSF 1200 μg, 1500 μg, and continuous rhG-CSF (Jisaixin) 5 μg/kg during the upfront two chemotherapeutic intermissions following treatment with docetaxel (T) 75 mg/m^2^ intravenously every 3 weeks, epirubicin (E) 75 mg/m^2^ intravenously every 3 weeks, with or without cyclophosphamide (C) 500 mg/m^2^ intravenously every 3 weeks. The experimental agent-rHSA/G-CSF-was given the first subcutaneous injection on the 3rd day after chemotherapy, followed by the second injection with an interval of 96 h., and the third administration was in the investigators’ choice depending on the dynamic monitored ANC.

### Efficacy and pharmacodynamic assessments

The primary efficacy endpoint of phase 2/2b trials was the duration of agranulocytosis (DOA) during the first chemotherapeutic intermission. The secondary endpoints were the DOA after the second chemotherapeutic performance, the length of the period until ANC climbing up to 2 × 10^9^/L after the initial peak following rHSA/G-CSF treatment, the dynamic ANC, the incidence and duration of FN, and the therapeutic course of prescribed antibiotics.

The count of WBC and neutrophils were dynamically monitored, as stipulated by protocol, in the course of therapeutic administration. To weaken the effect from confounders, permitted concomitant medications were limited to scheduled chemotherapeutics, antiemetic agents or other premedication at the discretion of the physician. Prophylactic corticosteroids or non-narcotic analgesics were not permitted unless undisciplinable allergies or pains occurred. Do not use antibiotics prophylactically. Antibiotics only were used in the case of grade 4 neutropenia lasting more than 3 days, neutropenia fever, definite infection, or axillary temperature ≥ 38.0 °C, and infectious fever that cannot be ruled out.

### Pharmacokinetic assessments

Plasma and urine rHSA/G-CSF concentrations were determined using a validated sandwich enzyme-linked immunosorbent assay. In single dosage, blood samples were collected before rHSA/G-CSF delivery and at 1, 2, 6, 12, 24, 48, 72, 96, 120, 144, 192, and 240 h after administration. In double dosage, blood samples were collected before rHSA/G-CSF delivery of the first cycle and at 1, 2, 6, 12, 24, 48, 72, 96 after first administration, and before the second delivery and 1, 2, 6, 12, 24, 48, 72, 96, 120, 144, 192 and 240 h after the second administration. PK parameters included maximum plasma concentration (C_max_), time to attain maximum plasma concentration (T_max_), area under plasma concentration-time curve (AUC), half-life (t_1/2_), apparent volume of distribution (Vz), clearance (CL), and mean residence time (MRT), the analysis of which performed by DAS 3.2.6 software using the non-compartmental method.

### Safety assessments

Patients were followed for adverse events (AEs) in systemic symptoms, physical signs, complete blood count, biochemistry, urinalysis, and electrocardiogram at screening and scheduled time point post dose. Safety was assessed by a statistical description of frequency and severity of AEs, characterized as mild, moderate, or severe. Serious AEs (SAEs) were defined as the AEs that resulted in death, hospitalization, the prolonged period of hospitalization, persistent or significant disability or incapacity, congenital anomaly or birth defect, or AEs that were otherwise medically important.

In this study, agranulocytosis or grade 4 neutropenia was defined as ANC < 0.5 × 10^9^/L, neutropenia was ANC ≤ 1.5 × 10^9^/L, and leukopenia was WBC ≤ 3 × 10^9^/L. FN was regarded as single oral temperature measurement > 38.3 °C (axilla temperature ≥ 38.0 °C), or oral temperature measurement > 38 °C lasting for at least 1 h, in company with neutropenia. DLT was referred to clinically significant AEs which were considered as associated with the experimental agent by investigators, including grade ≥ 2 hepatic or renal toxicity, grade ≥ 3 nonhematologic toxicity, and grade ≥ 4 hematologic toxicity lasting ≥7 days. All the classifications of AEs categories and grad were followed Common Terminology Criteria for Adverse Events (CTCAE) version 4.0.

### Statistical analysis

The analysis of variance and the Kruskal-Wallis test were used to test for divergence across groups. Pairwise differences were inspected using the *t* test or the Wilcoxon rank sum test as appropriate. Statistical tests were two-sided with a significant level set at 0.05. All statistical analyses were performed using the SPSS software version 16.0.

## Results

### Dose escalation – phase 1b

**Fig. 1:**
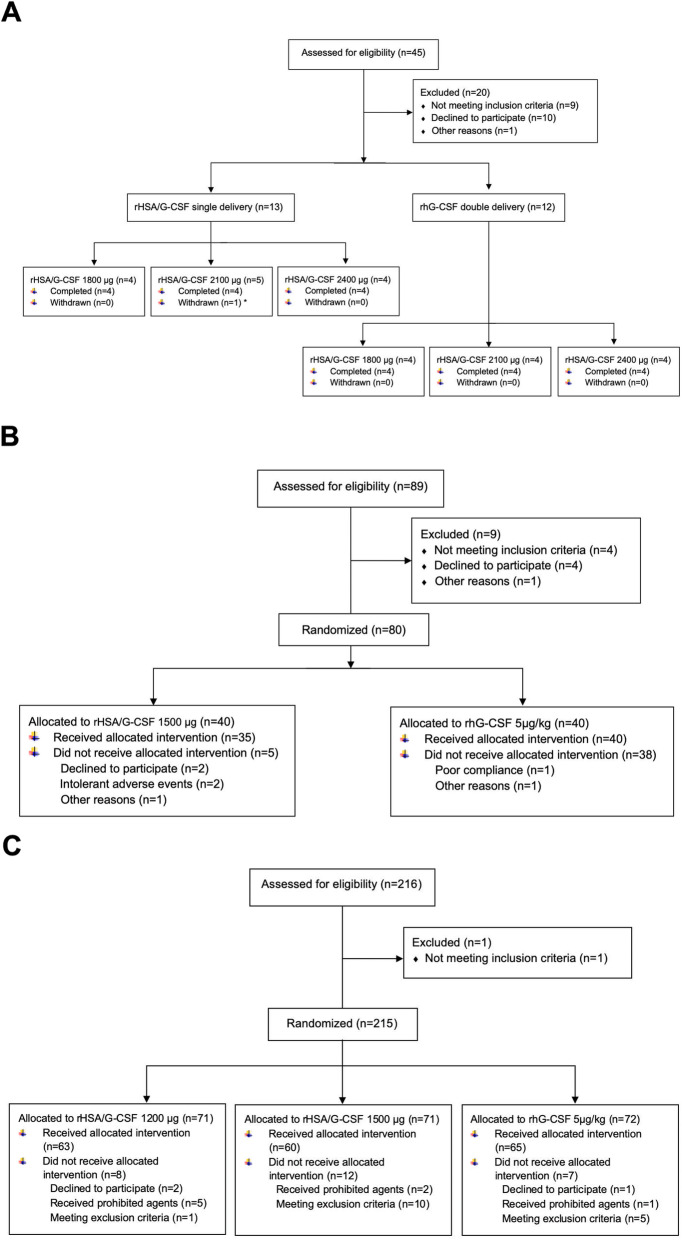
Flow diagram of enrollment, allocation, follow-up, and analysis of patients included in phase 1b (**a**), phase 2b (**b**), and phase 2 (**c**). * one patient was withdrawn for the occurrence of grade 4 neutropenia

A total of 29 patients in three research centers were enrolled into the phase 1b study, which included 13 patients in the single-dose proportion and 12 patients in the multi-dose part (Fig. 1a). The mean age was 47.1 ± 11.38 years (Table S1 in the Online Resource). In this proportion, the safety, pharmacokinetics, pharmacodynamics within an escalating dose of rHSA/G-CSF, from 1800 μg to 2400 μg, were successively assessed.

Results from safety analysis demonstrated that a total of 37 AEs occurred in 12 out of 13 (92.3%) subjects enrolled in single-dose part while 29 AEs were observed in 8 out of 12 (66.7%) participants involved in multi-dose cohorts. The most commonly reported AEs of any grade were increased alanine aminotransferase (ALT) (21.6%), anemia (18.9%), increased aspartic aminotransferase (AST) (10.8%), and just 3 treatment-related AEs were recorded including ostealgia and eye edema up to grade 2 (Table S2 in the Online Resource). No treatment-related SAEs and DLT were recorded in the course of dose escalation of rHSA/G-CSF. Moreover, no apparent dosage effect was detected on the risk of treatment-related AEs.

**Fig. 2:**
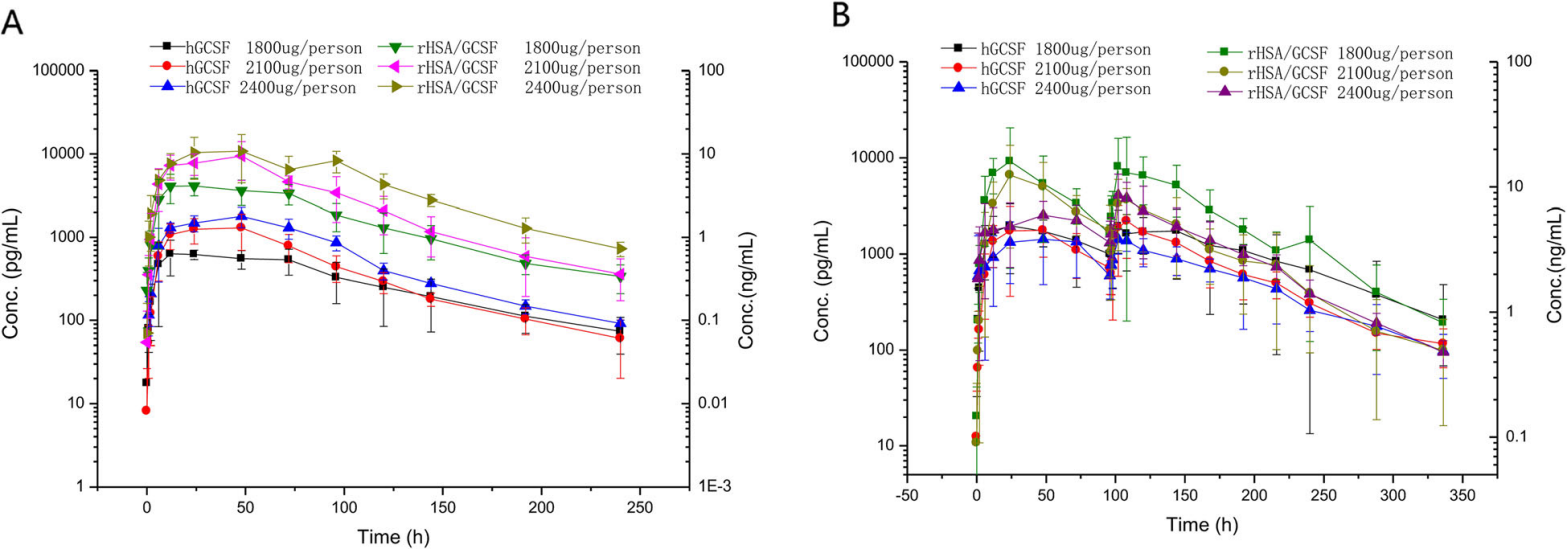
Pharmacokinetics analysis of single dosage of rHSA/G-CSF (**a**) and double dosage of rHSA/G-CSF (**b**) from dose-escalation phase 1b trial

**Fig. 3:**
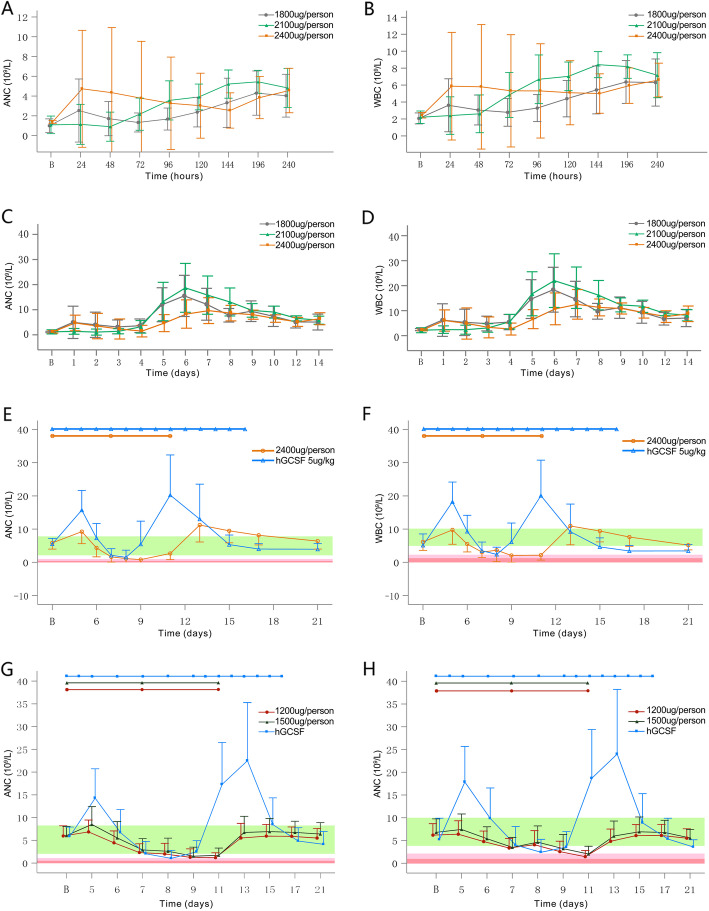
Dynamic measures of ANC and WBC in phase 1b (**a-d**), phase 2b (**e-f**), and phase 2 (**g-h**). The delivery pattern of rHSA/G-CSF in randomized trials were shown on the top of the corresponding curves, while the ranges of grade 4, grade 3, and normalization were demonstrated in accordance with the corresponding values

The mean levels of rHSA/G-CSF in escalating dose from PK analysis of phase 1b over time were shown in Fig. 2. For the single-dose part, two distinct pharmacokinetic phases including a rapid initial increase and a slow decline phase were identified in all dose cohorts. The average concentration of G-CSF observed increased with the dose escalation of rHSA/G-CSF and reached the peak within 12–48 h. Then the blood concentration of rhG-CSF decreased until 240 h after the administration and fell to the lower limit of quantification (39.1 pg/mL). In multi-dose proportion, the mean plasma concentrations-time curve presented double peaks, consistent with double delivery of the studied agent, and no solid evidence for dose-dependence effect. Urine rHSA/G-CSF concentration was undetectable in patients at any dose levels of the study. Calculated PK parameters for 1800 μg, 2100 μg, and 2400 μg group were summarized in (Table S3 in the Online Resource). This proportion of findings suggested that AUC and Cmax were increased in accordance with the delivery of dose escalation, namely, a 1.3-fold increase in dose from 1800 to 2100 μg resulted in approximately a 2.4-fold increase in Cmax. However, there was no dose-associated trend detected, considering, no significant differences in t_1/2_ exhibited within 60–65 h and Tmax was relatively consistent in the range of 28–39 h at doses of 1800 to 2400 μg. The elimination of rHSA/G-CSF tended to retard following the course of dose escalation (i.e. decreasing Cl/F). The statistical analysis of derived PK parameters indicated against dosage effect but the inherent variability from enrolled participants. No cycle-to-cycle accumulation (i.e. AUC _(0-t)_, AUC _(0-∞)_, *P* > 0.05) were shown in both the single-dose and multi-dose cohorts (Table S4 in the Online Resource). The corresponding dynamic measures of ANC and WBC were demonstrated in (Fig. 3a-d).

This stage of study, in sum, recommended that the double dosage regimens of rHSA/G-CSF 2400 μg had tolerable safety profiles on the basis of dose-independent toxicities, and promising efficacy in favor of the management for neutropenia induced by chemotherapy in clinical practice.

### Dose expansion – phase 2b

A total of 80 patients, including 40 patients in the 2400 μg group and 40 patients in the control group, were enrolled from 10 research centers and randomly allocated (Fig. 1b). Demographics and clinical characteristics at baseline were balanced across treatment groups (Table S2 in the Online Resource).

In this phase 2b proportion, the mean DOA was 2.1 ± 1.72 days in rHSA/G-CSF 2400 μg group and 0.8 ± 1.06 days in the rhG-CSF control group, respectively, which was statistically different (*P* = 0.002) and failed to be advantageous over rhG-CSF. This result was consistent with those from stratified analysis of chemotherapy protocol. However, the motivation of ANC tended to be moderate with a steady trend exhibited in the dynamic curve of neutrophils increased (Fig. 3e-f). Safety analysis revealed that there were 39 AEs occurred in 13 (32.5%) patients of 2400 μg group and 39 AEs occurred in 11 (27.5%) patients. The most frequently occurring AEs in 2400 μg were increased ALT (43.6%) and increased AST (23.1%). All the AEs were reportedly in mild or moderate intensity and possibly or not clinically significant, and were well manageable (Table S5 in the Online Resource).

### Comparative efficacy and safety – phase 2

A total of 215 breast cancer patients were recruited from 17 institutions across the nation and randomly allocated to three cohorts in phase 2 trial, of which 71 participants received rHSA/G-CSF 1200 μg, 71 participants received rHSA/G-CSF 1500 μg, and 72 participants were treated with rhG-CSF 5 μg/kg, respectively (Fig. 1c). The mean age was 40.8 years, and the population demographics at baseline were well balanced (Table S1 in the Online Resource).

The DOA following the first receipt of chemotherapy was observed as 1.59 ± 1.59 days in rHSA/G-CSF 1200 μg, 1.14 ± 1.35 days in rHSA/G-CSF 1500 μg, and 1.07 ± 0.97 days in rhG-CSF, respectively. This time of duration from patients in rHSA/G-CSF 1500 μg was proven to be comparable with that of the controlled group (*P* = 0.712), while patients who received rHSA/G-CSF 1200 μg were suggested to suffer a prolonged DOA than those in both rhG-CSF cohort (*P* = 0.019) and rHSA/G-CSF 1500 μg (*P* = 0.046). The mean DOA after the second chemotherapeutic performance was 0.85 ± 1.35 days in rHSA/G-CSF 1200 μg, 0.50 ± 0.96 days in rHSA/G-CSF 1500 μg, and 0.44 ± 0.77 days in rhG-CSF, respectively, and the results indicated that no statistical differences were detected between rHSA/G-CSF 1500 μg and rhG-CSF as well as rHSA/G-CSF 1500 μg and rHSA/G-CSF 1200 μg. The incidences of febrile neutropenia were 23.9, 6.9, and 1.11% in rHSA/G-CSF 1200 μg, rHSA/G-CSF 1500 μg, and rhG-CSF group with statistical differences detected between rHSA/G-CSF 1500 μg and the other two cohorts, indicating that patients preventively receiving rHSA/G-CSF 1500 μg suffered the least risk of this detrimental complication following cytotoxic agents. However, no statistical differences of the durative period of febrile neutropenia in addition to the rate of antibiotic administration were demonstrated among the three groups. Besides, for the time before ANC ascending to 2 × 10^9^/L from the first peak and the dynamic count of neutrophils, a steady trend in the growth of ANC was observed which revealed a moderate stimulus intensity for ANC provided by rHSA-G-CSF, although this recorded period of both rHSA/G-CSF 1200 μg and 1500 μg was prolonged than that of rhG-CSF (Fig. 3g-h).

The analysis of PK showed that the rhG-CSF concentration in the 1200 μg or 1500 μg test group was lower than that in the control group, and its distribution and elimination were also slower, presenting the characteristics of long-acting drugs. The half-life of 1200 μg and 1500 μg group after subcutaneously administered to the subjects was about 24-80 h that was significantly longer than that of the control drug with 7-16 h. There was no significant difference in the half-life and other kinetic parameters (such as Cmax and AUC) between the two-dose groups compared with the previous test (the results of drug metabolism on phase 1 clinical trial of drug dose climbing test).

Safety profiles revealed that 36 AEs in total occurred in 25 (11.6%) patients including 10 AEs in 6 (8.5%) patients of 1200 μg group, 9 AEs in 8 (11.1%) patients of 1500 μg group, and 17 AEs in 11 (15.3%) patients of the control group. The leading AEs in 1200 μg and 1500 μg were ostealgia (21.1%), weak (15.8%), fever (15.8%), and anemia (15.8%), while the frequently occurring AEs in rhG-CSF group were backache (27.3%), fever (18.2%) and thrombocytopenia (9.1%). No statistical difference was suggested, and no treatment-related SAEs were observed in the three cohorts (Table S5 in the Online Resource). On balance, rHSA/G-CSF was well tolerated among breast cancer with the receipt of chemotherapy in the upfront stage.

In general, the dosage regimens based on rHSA/G-CSF 1500 μg have comparable efficacy and safety profiles with rhG-CSF, which tended to be the recommended preventive management for serious neutropenia, especially following anthracycline-containing chemotherapy, in clinical practice. Besides, in this proportion, it was shown that the primary outcome measure of the rHSA/G-CSF 1200 μg cohort was statistically inferior to that of a higher dosage regimen based on rHSA/G-CSF 1500 μg, indicating a promising dose-dependent effect from the experimental agent. This compelling finding facilitated the design and introduction, to assess the clinical safety and response from dose escalation, of phase 1b trial at the next stage.

## Discussion

This study, comprising three stages of trials, initially confirmed the clinical response and safety profiles of rHSA/G-CSF among patients with breast cancer in terms of both prophylactical and adjuvant management of serious neutropenia following anthracycline-based chemotherapy. In comparisons with rhG-CSF, this agent with novel design and preparation could produce a steady stimulus motivation of neutrophils with relatively more moderate intensity, weakening the detrimental effects on patients from cytokines storm induced by a blasting release of G-CSF [26], and constitute a proportion of approachable therapeutics for chemotherapy-induced toxicities.

Randomized studies suggested that the double delivery of rHSA/G-CSF 1500 μg was a non-inferior treatment, in comparison with rhG-CSF, for breast cancer patients following chemotherapy based on TEC or TE regimens. On the basis of considerable efficacy, this kind of delivery frequency with two times during a chemotherapeutic intermission was more approachable for patients than that of rhG-CSF with continuous injection. Moreover, the overall dose of rHSA/G-CSF with one cycle of chemotherapy was fairly less than the equivalent agents based on G-CSF, such as rhG-CSF and PEG-rhG-CSF. It meant that the overall dose of PEG-rhG-CSF needed for one cycle of chemotherapy is 6 mg and the rhG-CSF cumulative dose is 4.2 mg (14 times per cycle) [27], whereas the dosage of rHSA/GCSF was expected to be 3–4.5 mg/person, namely, equivalent of 0.75 to 1.1 mg conventional rhG-CSF preparation. This characteristic constituted the foundation of more safety and fewer toxicities induced by a blast of G-CSF infusion. Furthermore, the outcomes from the dynamic record of ANC demonstrated a steady ascending curve after the delivery of rHSA/G-CSF with few risks of grade 4 neutropenia, and the ANC at the end of the corresponding intermission was within the normalized boundary. Notably, the ANC on the 12th day was recovered in normalization and further remained standard with a minor fluctuation. This moderate measure of ANC could facilitate the smooth completion of anthracycline-containing chemotherapy and potentially provide benefits for patients with early-stage malignancies. Although the chemotherapeutic regimen administrated on participants might not be routinely recommended for breast cancer in international practice, it has been widely adopted with confirmative efficacy for breast cancer patients in China [28–31]. Collectively, the efficacious profile observed in rHSA/G-CSF was promising, of which the population generality deserved prospective validation.

Through the initial assessment of the comparative phase 2 trial, the rHSA/G-CSF 1200 μg exhibited a prolonged DOA than rHSA/G-CSF 1500 μg, which revealed a dose effect on the stimulus motivation of neutrophils following anthracycline-containing chemotherapy. However, the following trials reported against this effect from regimens based on a higher dose at 2400 μg, which failed to provide a noninferiority response in terms of shortening DOA similar to 1500 μg in phase 2 study. This phenomenon indicated that the motivation of hematopoietic function might not synchronize with dose escalation of G-CSF agents, which was consistent with several studies conducted previously [32, 33]. It seemed that the G-CSF preparation dose within a rationale dosage frame would be a crucial proportion for trials to explore.

Safety analysis revealed that rHSA/GCSF was well tolerated, and no serious toxicities were observed with the administration on breast cancer patients receiving myelosuppressive chemotherapy. The majority of AEs observed in this study were moderate and well-managed, even the foremost proportion of abnormalities from laboratory examinations were considered as far from treatment-related. On the basis of advantages in pharmacodynamic and pharmacokinetic assessments, patients treated with the delivery of rHSA-G-CSF were less vulnerable to transient leukemia symptoms, such as ostealgia and fever, which could contribute to superior treatment compliance. Of note, an approximate 20% incidence of thrombocytopenia occurred in the interventional group with consistency in the previous trials [27, 33]. Howbeit this mechanism remained undetermined, this concomitant phenomenon could be the result of crosstalk over signaling pathways of granulocyte and platelets at the primitive differentiation inception [34].

Randomized control trials comparing the pegylated G-CSF preparation and Balugrastim, which was designed and developed in the identical molecular structure, have reported a noninferiority efficacy and comparable safety profiles in breast cancer patients with the receipt of chemotherapy [27, 35]. In fact, at the beginning of the trials, the pegylated G-CSF preparation was not officially approved of application in China, thus we considered rhG-CSF as paralleling control. In the approaching phase 3 trial, the corresponding cohort would be established and accomplish a head-to-head comparative trial. More solid evidence will be provided for clinical practice in the foreseeable future.

In this study, rHSA/GCSF, as a novel preparation, was proven to be efficacious and well-tolerated in the course of prevention and management of agranulocytosis from breast cancer patients following upfront chemotherapy. With the comparable efficacy and safety profiles with rhG-CSF, this kind of injectable medication with both a less frequency and a lower expense of application could facilitate patients to receive the delivery of both chemotherapy and corresponding treatment for associated side effects in clinical practice.

## Supplementary Information

**Additional file 1 **: **Table S1**. Population demographics and baseline characteristics in this study. **Table S2**. Safety profiles of patients enrolled in dose-escalation study. **Table S3**. Pharmacokinetics parameters calculated from phase 1b trial. **Table S4**. Pharmacokinetics analysis from dose-escalation phase 1b trial. **Table S5**. Safety profiles of patients included in randomized studies

## Data Availability

The datasets used and/or analyzed during the current study are available from the corresponding author on reasonable request.

## Abbreviations

FN: Febrile neutropenia
G-CSF: Recombinant granulocyte-colony stimulating factor
PEG: Polyethylene glycol
HAS: Human serum albumin
rHSA/G-CSF: Recombinant human serum albumin /granulocyte colony-stimulating factor
PK: Pharmacokinetics
PD: Pharmacodynamics
ANC: Absolute neutrophil count
WBC: White blood cell
DLT: Dose-limiting toxicity
DOA: Agranulocytosis
C_max_: Maximum plasma concentration
T_max_: Maximum plasma concentration
AUC: Area under plasma concentration-time curve
t_1/2_: Half-life
Vz: Volume of distribution
CL: Clearance
MRT: Mean residence time
AE: Adverse events
SAEs: Serious adverse events
CTCAE: Common Terminology Criteria for Adverse Events
ALT: Alanine aminotransferase
AST: Aspartic aminotransferase
