# Supplementary material for: Randomized and dose-escalation trials of recombinant human serum albumin /granulocyte colony-stimulating factor in patients with breast cancer receiving anthracycline-containing chemotherapy
Source: BMC Cancer. 2021 Mar 31;21:341. doi: 10.1186/s12885-021-08093-z (PMC8010964; doi:10.1186/s12885-021-08093-z)
Supplement: Supplementary file 1 — Additional file 1 : Table S1. Population demographics and baseline characteristics in this study. Table S2. Safety profiles of patients enrolled in dose-escalation study. Table S3. Pharmacokinetics parameters calculated from phase 1b trial. Table S4. Pharmacokinetics analysis from dose-escalation phase 1b trial. Table S5. Safety profiles of patients included in randomized studies [file 12885_2021_8093_MOESM1_ESM.docx]

Online Resource

Randomized and dose-escalation trials of recombinant human serum albumin /granulocyte colony-stimulating factor in patients with breast cancer receiving chemotherapy

Journal Name: Annals of Hematology

Shanshan Chen^1^*, Yiqun Han^1^*, Quchang Ouyang^2^, Jianguo Lu^3^, Qingyuan Zhang^4^, Shun'e Yang^5^, Jingfen Wang^6^, Haixin Huang^7^, Hong Liu^8^, Zhimin Shao^9^, Hui Li^10^, Zhendong Chen^11^, Sanyuan Sun^12^, Cuizhi Geng^13^, Junguo Lu^14^, Jianwei Sun^15^, Jiayu Wang^1#^, Binghe Xu^1#^

1. Department of Medical Oncology, National Cancer Center/ National Clinical Research Center for Cancer/ Cancer Hospital, Chinese Academy of Medical Sciences and Peking Union Medical College, Beijing, China

2. Department of Breast Cancer Medical Oncology, Hunan Cancer Hospital, Changsha, Hunan, China

3. Department of General Surgery, Xi'an Tangdu Hospital, Xi’an, Shanxi, China

4. Department of Oncology, Harbin Medical University Cancer Hospital, Harbin, Heilongjiang, China

5. Department of Breast Cancer and Lymphoma, The Affiliated Tumor Hospital of Xinjiang Medical University, Urumqi, Xinjiang, China

6. Department of Internal Medicine, Linyi Tumor Hospital, Linyi, Shandong, China

7. Department of Oncology, The Fourth Affiliated Hospital of Guangxi Medical University, Liuzhou, Guangxi, China

8. Department of Breast Surgery, National Clinical Research Center for Cancer, Tianjin Medical University Cancer Institute and Hospital, Tianjin, China

9. Department of Breast Surgery, Fudan University Shanghai Cancer Center, Shanghai, China

10. Department of Breast Surgery, Sichuan Province Tumor Hospital, Chengdu, Sichuan, China

11. Department of Medical Oncology, The Second Affiliated Hospital of Anhui Medical University, Hefei, Anhui, China

12. Department of Medical Oncology, Central Hospital of Xuzhou, the Cancer Institute of Southeast University, Xuzhou, Jiangsu, China

13. First Department of Surgery, The Fourth Hospital of Hebei Medical University, Shijiazhuang, Hebei, China

14. Department of Medical Oncology, Nantong Tumor Hospital, Nantong, Jiangsu, China

15. Department of Tumor, Yunnan First People’s Hospital, Kunming, Yunnan, China

*These authors contributed equally to this study

^#^Corresponding authors:

Binghe Xu, MD, Department of Medical Oncology, National Cancer Center/National Clinical Research Center for Cancer/Cancer Hospital, Chinese Academy of Medical Sciences and Peking Union Medical College. No. 17 Panjiayuan Nanli, Chaoyang District, Beijing, 100021, China. Fax: +86-87788120 Email: xubingheBM@163.com

Jiayu Wang, MD, Department of Medical Oncology, National Cancer Center/Cancer Hospital, Chinese Academy of Medical Sciences and Peking Union Medical College. No. 17 Panjiayuan Nanli, Chaoyang District, Beijing, 100021, China. Fax: +86-87788120 Email: wangjiayu8778@sina.com

Table S1. Population demographics and baseline characteristics in this study

| Variables | Phase 1b | | | | | | |  | Phase 2 | | |  | Phase 2b | |
| --- | --- | --- | --- | --- | --- | --- | --- | --- | --- | --- | --- | --- | --- | --- |
|  | Single dose | | |  | Double dose | | |  |  | | |  | |  |
|  | 1800 μg | 2100 μg | 2400 μg |  | 1800 μg | 2100 μg | 2400 μg |  | 1200 μg | 1500 μg | rhG-CSF |  | 2400 μg | rhG-CSF |
| N | 4 | 5 | 4 |  | 4 | 4 | 4 |  | 71 | 72 | 72 |  | 39 | 40 |
| Age, years (mean ± SD) | 54.5±3.11 | 39.6±14.36 | 49.0±7.87 |  | 41.8±8.96 | 42.0±10.86 | 38.8±6.55 |  | 47.6±10.40 | 47.2±8.20 | 47.0±9.22 |  | 48.8±10.10 | 45.7±8.22 |
| Height, cm (mean ± SD) | 157.0±2.45 | 156.8±5.72 | 160.0±4.97 |  | 154.0±2.71 | 155.0±4.16 | 157.3±11.62 |  | 158.2±5.43 | 157.6±5.60 | 156.2±4.81 |  | 157.2±5.078 | 157.4±5.77 |
| Weight, kg (mean ±SD) | 56.5±10.97 | 60.0±11.68 | 59.0±8.16 |  | 49.3±2.22 | 64.5±7.05 | 56.3±10.37 |  | 60.5±9.20 | 59.7±8.91 | 57.1±9.30 |  | 59.4±9.77 | 57.7±8.88 |
| BMI, kg/m^2^ (mean ± SD) | 23.0±4.18 | 24.3±2.99 | 23.0±1.83 |  | 20.8±0.19 | 26.8±1.74 | 22.6±0.86 |  | 24.1±3.00 | 24.1±3.30 | 23.4±3.51 |  | 24.0±3.54 | 23.3±3.42 |
| Han Nationality, n (%) | 4 (100) | 5 (100) | 4 (100) |  | 4 (100) | 3 (75) | 4 (100) |  | 61 (85.9) | 68 (94.4) | 67 (93.1) |  | 37 (94.9) | 38 (95.0) |
| Female, n (%) | 4 (100) | 5 (100) | 4 (100) |  | 4 (100) | 4 (100) | 4 (100) |  | 71 (100) | 72 (100) | 72 (100) |  | 39 (100.0) | 40 (100.0) |
| PS score, n (%) |  |  |  |  |  |  |  |  |  |  |  |  |  |  |
| 0 | 4 (100) | 3 (60) | 4 (100) |  | 2 (50) | 2 (50) | 4 (100) |  | 56 (78.9) | 49 (68.1) | 47 (65.3) |  | 24 61.5) | 25 (62.5) |
| 1 | 0 (0) | 2 (40) | 0 (0) |  | 2 (50) | 2 (50) | 0 (0) |  | 15 (21.1) | 23 (31.9) | 25 (34.7) |  | 15 (38.5) | 15 (37.5) |
| Chemotherapy exposure of the first cycle, n (%) | | | | | | | | | | | | | | |
| TE | 0 | 0 | 1 (25) |  | 0 | 0 | 0 |  | 33 (46.5) | 34 (47.2) | 33 (45.8) |  | 17 (43.6) | 20 (50.0) |
| TEC | 2 (50) | 2 (40) | 0 |  | 0 | 2 (50) | 3 (75) |  | 38 (53.5) | 38 (52.8) | 38 (52.8) |  | 22 (56.4) | 20 (50.0) |
| others | 2 (50) | 3 (60) | 3 (75) |  | 4 (100) | 2 (50) | 1 (25) |  | 0 | 0 | 0 |  | 0 | 0 |
| Chemotherapy exposure of the second cycle, n (%) | | | | | | | | | | | | | | |
| TE | __ | __ | __ |  | 0 | 0 | 0 |  | 32 (46.4) | 32 (45.7) | 32 (45.7) |  | 16 (45.7) | 19 (50.0) |
| TEC | __ | __ | __ |  | 0 | 2 (50) | 3 (75) |  | 37 (53.6) | 38 (54.3) | 36 (51.4) |  | 19 (54.3) | 19 (50.0) |
| others | __ | __ | __ |  | 4 (100) | 2 (50) | 1 (25) |  | 0 | 0 | 0 |  | 0 | 0 |
| Clinical stage of breast cancer | | | | | | | | | | | | | | |
| 0, n (%) | 0 (0) | 0 (0) | 0 (0) |  | 0 (0) | 0 (0) | 0 (0) |  | 0 (0) | 0 (0) | 0 (0) |  | 0 (0) | 0 (0) |
| I, n (%) | 0 (0) | 0 (0) | 0 (0) |  | 1 (25) | 0 (0) | 0 (0) |  | 7 (10.3) | 3 (4.5) | 8 (11.4) |  | 0 (0) | 4 (10.3) |
| II, n (%) | 0 (0) | 3 (60) | 0 (0) |  | 1 (25) | 2 (50) | 0 (0) |  | 26 (38.2) | 22 (32.8) | 20 (28.6) |  | 18 (48.6) | 14 (35.9) |
| III, n (%) | 3 (75) | 1 (20) | 1 (25) |  | 1 (25) | 2 (50) | 3 (60) |  | 28 (41.2) | 32 (47.8) | 31 (44.3) |  | 15 (40.5) | 14 (35.9) |
| IV, n (%) | 1 (25) | 1 (20) | 2 (50) |  | 1 (25) | 0 (0) | 1 (25) |  | 7 (10.3) | 10 (14.9) | 11 (15.7) |  | 4 (10.8) | 7 (17.9) |
| missing, n (%) | 0 (0) | 0 (0) | 1 (25) |  | 0 (0) | 0 (0) | 0 (0) |  | 9 (12.7) | 6 (8.3) | 6 (8.3) |  | 2 (5.1) | 1 (2.5) |

Table S2. Safety profiles of patients enrolled in dose-escalation study

| Event, n % | Phase Ib | | | | | | | | | | | | | | | | | | |
| --- | --- | --- | --- | --- | --- | --- | --- | --- | --- | --- | --- | --- | --- | --- | --- | --- | --- | --- | --- |
|  | Part 1 | | | | | | | | |  | Part 2 | | | | | | | | |
|  | 1800 μg | | | 2100 μg | | | 2400 μg | | |  | 1800 μg | | | 2100 μg | | | 2400 μg | | |
| Grade | I | II | III | I | II | III | I | II | III |  | I | II | III | I | II | III | I | II | III |
| All kinds of inspection | | | | | | | | | | | | | | | | | | | |
| Alanine aminotransferase increased | 4(30.8) | 1(16.7) | 1(100) | 1(33.3) | 1(33.3) | 0 | 0 | 0 | 0 |  | 0 | 0 | 0 | 1(16.7) | 0 | 0 | 3(33.3) | 1(9.1) | 0 |
| Aspartic aminotransferase increased | 2(15.4) | 1(16.7) | 0 | 1(33.3) | 0 | 0 | 0 | 0 | 0 |  | 0 | 0 | 0 | 1(16.7) | 0 | 0 | 2(22.2) | 0 | 0 |
| Hyperhemoglobinemia | 0 | 0 | 0 | 0 | 0 | 0 | 3(37.5) | 0 | 0 |  | 0 | 1(100) | 0 | 0 | 0 | 0 | 0 | 0 | 0 |
| Thrombocytopenia | 0 | 0 | 0 | 0 | 0 | 0 | 1(12.5) | 0 | 0 |  | 0 | 0 | 0 | 0 | 0 | 0 | 0 | 0 | 0 |
| Gastrointestinal disorders | | | | | | | | | | | | | | | | | | | |
| Nausea | 1(7.7) | 0 | 0 | 0 | 0 | 0 | 0 | 0 | 0 |  | 0 | 0 | 0 | 1(16.7) | 0 | 0 | 1(11.1) | 1(9.1) | 0 |
| Stomachache | 1(7.7) | 0 | 0 | 0 | 0 | 0 | 0 | 0 | 0 |  | 0 | 0 | 0 | 1(16.7) | 0 | 0 | 0 | 0 | 0 |
| Diarrhea | 0 | 1(16.7) | 0 | 0 | 0 | 0 | 0 | 0 | 0 |  | 0 | 0 | 0 | 1(16.7) | 0 | 0 | 0 | 0 | 0 |
| Emesis | 0 | 0 | 0 | 0 | 0 | 0 | 0 | 0 | 0 |  | 0 | 0 | 0 | 0 | 0 | 0 | 1(11.1) | 1(9.1) | 0 |
| Diseases of the blood and lymphatic system | | | | | | | | | | | | | | | | | | | |
| Anemia | 1(7.7) | 2(33.3) | 0 | 0 | 1(33.3) | 0 | 2(25.0) | 1(33.3) | 0 |  | 0 | 0 | 0 | 0 | 0 | 0 | 0 | 6(54.5) | 0 |
| Respiratory diseases | | | | | | | | | | | | | | | | | | | |
| Cough | 1(7.7) | 0 | 0 | 0 | 0 | 0 | 0 | 0 | 0 |  | 0 | 0 | 0 | 1(16.7) | 0 | 0 | 0 | 0 | 0 |
| Ocular disease |  |  |  |  |  |  |  |  |  |  |  |  |  |  |  |  |  |  |  |
| Swelling of eye | 0 | 0 | 0 | 0 | 0 | 0 | 0 | 1(33.3) | 0 |  | 0 | 0 | 0 | 0 | 0 | 0 | 0 | 0 | 0 |
| Metabolic and nutritional diseases | | | | | | | | | | | | | | | | | | | |
| Hyponatremia | 1(7.7) | 0 | 0 | 0 | 0 | 0 | 0 | 0 | 0 |  | 0 | 0 | 0 | 0 | 0 | 0 | 0 | 0 | 0 |
| Nervous system disease | | | | | | | | | | | | | | | | | | | |
| Headache | 0 | 0 | 0 | 0 | 0 | 0 | 1(12.5) | 0 | 0 |  | 0 | 0 | 0 | 0 | 0 | 0 | 0 | 0 | 0 |
| Dizzy | 0 | 0 | 0 | 0 | 0 | 0 | 1(12.5) | 0 | 0 |  | 0 | 0 | 0 | 0 | 0 | 0 | 1(11.1) | 1(9.1) | 0 |
| Insomnia | 0 | 0 | 0 | 0 | 0 | 0 | 0 | 0 | 0 |  | 0 | 0 | 0 | 0 | 0 | 0 | 0 | 0 | 1(100) |
| Musculoskeletal and connective tissue diseases | | | | | | | | | | | | | | | | | | | |
| Backache | 0 | 0 | 0 | 1(33.3) | 1(33.3) | 0 | 0 | 1(33.3) | 0 |  | 0 | 0 | 0 | 0 | 0 | 0 | 0 | 0 | 0 |
| Musculoskeletal pain | 0 | 0 | 0 | 0 | 0 | 0 | 0 | 0 | 0 |  | 0 | 0 | 0 | 0 | 0 | 0 | 1(11.1) | 1(9.1) | 0 |
| Systemic diseases and various reactions at the site of administration | | | | | | | | | | | | | | | | | | | |
| Weak | 1(7.7) | 0 | 0 | 0 | 0 | 0 | 0 | 0 | 0 |  | 0 | 0 | 0 | 0 | 0 | 0 | 0 | 0 | 0 |
| Fever | 1(7.7) | 1(16.7) | 0 | 0 | 0 | 0 | 0 | 0 | 0 |  | 0 | 0 | 0 | 0 | 0 | 0 | 0 | 0 | 0 |
| Infectious and infectious diseases | | | | | | | | | | | | | | | | | | | |
| Urinary tract infection | 0 | 0 | 0 | 0 | 0 | 0 | 0 | 0 | 0 |  | 1(100) | 0 | 0 | 0 | 0 | 0 | 0 | 0 | 0 |

Table S3. Pharmacokinetics parameters calculated from phase 1b trial

| Dose | AUC _(0-240 h)_ | AUC_INF_ | C_max_ | t_1/2_ | T_max_ | Vz/F | Cl/F |
| --- | --- | --- | --- | --- | --- | --- | --- |
|  | ng·h /mL | ng·h /mL | ng/mL | (h) | (h) | L | L/h |
|  | Mean±SD | Mean±SD | Mean±SD | Mean±SD | Mean±SD | Mean±SD | Mean±SD |
| 1800μg | 73.75±23.88 | 80.58±25.68 | 0.76±0.19 | 65.59±10.95 | 39±26.61 | 2381.65±1170.57 | 24.33±8.55 |
| 2100μg | 100.53±31.70 | 113.69±35.34 | 1.39±0.55 | 65.98±22.47 | 28.8±18.2 | 1782.68±418.19 | 20.03±6.87 |
| 2400μg | 166.66±23.55 | 174.67±22.56 | 1.83±0.43 | 60.32±6.15 | 39±18 | 1220.46±271.27 | 13.94±2.06 |

Table S4. Pharmacokinetics analysis from dose-escalation phase 1b trial

| Dosage regimens | Cmax (ng/mL) | AUC (0-t) (ng·h /mL) | AUCINF (ng·h /mL) |
| --- | --- | --- | --- |
| 1800μg, single dose | 0.76±0.17 | 73.75±20.66 | 80.58±22.24 |
| 1800μg, double dose | 2.75±0.99 | 224.09±141.91 | 243.98±164.06 |
| *P* value | 0.014 | 0.12 | 0.14 |
| 2100μg, single dose | 1.18±0.28 | 106.40±28.86 | 113.69±30.61 |
| 2100μg, double dose | 2.83±0.83 | 108.73±72.40 | 171.80±61.67 |
| *P* value | 0.012 | 0.96 | 0.43 |
| 2400μg, single dose | 1.83±0.37 | 166.66±20.39 | 174.67±19.54 |
| 2400μg, double dose | 1.93±0.40 | 124.59±37.40 | 133.61±40.39 |
| *P* value | 0.75 | 0.14 | 0.16 |

Table S5. Safety profiles of patients included in randomized studies

| Event, n % | Phase 2 | | | | | | | | |  | Phase 2b | | | | | |
| --- | --- | --- | --- | --- | --- | --- | --- | --- | --- | --- | --- | --- | --- | --- | --- | --- |
|  | 1200 μg | | | 1500 μg | | | rhG-CSF | | |  | 2400 μg | | | rhG-CSF | | |
| Grade | 1 | 2 | 3 | 1 | 2 | 3 | 1 | 2 | 3 |  | 1 | 2 | 3 | 1 | 2 | 3 |
| All kinds of inspection | | | | | | | | | | | | | | | | |
| Alanine aminotransferase increased | 0 | 0 | 0 | 0 | 0 | 0 | 0 | 0 | 0 |  | 12(41.4) | 3(60) | 2(40) | 7 (21.9) | 1(16.7) | 0 |
| Aspartic aminotransferase increased | 0 | 0 | 0 | 0 | 0 | 0 | 0 | 0 | 0 |  | 8(27.6) | 1(20) | 0 | 6(18.8) | 1(16.7) | 0 |
| Blood lactate dehydrogenase increased | 0 | 0 | 0 | 0 | 0 | 0 | 0 | 0 | 0 |  | 2(6.9) | 0 | 0 | 0 | 2(33.3) | 0 |
| Blood alkaline phosphatase increased | 0 | 0 | 0 | 0 | 0 | 0 | 0 | 0 | 0 |  | 2(6.9) | 0 | 0 | 0 | 0 | 0 |
| The percentage of lymphocytes decreased | 0 | 0 | 0 | 0 | 0 | 0 | 0 | 0 | 0 |  | 0 | 0 | 0 | 3(9.4) | 0 | 0 |
| Lymphocyte count increased | 0 | 0 | 0 | 0 | 0 | 0 | 0 | 0 | 0 |  | 0 | 0 | 0 | 1(3.1) | 0 | 0 |
| Blood bilirubin increased | 0 | 0 | 0 | 0 | 0 | 0 | 0 | 0 | 0 |  | 1(3.4) | 0 | 0 | 0 | 0 | 0 |
| Thrombocytopenia | 0 | 0 | 0 | 0 | 0 | 0 | 1(9.1) | 1(20) | 1(100) |  | 0 | 0 | 0 | 0 | 0 | 0 |
| Gastrointestinal disorders | | | | | | | | | | | | | | | | |
| Emesis | 0 | 0 | 0 | 0 | 0 | 0 | 0 | 0 | 0 |  | 1(3.4) | 0 | 0 | 0 | 0 | 0 |
| Diseases of the blood and lymphatic system | | | | | | | | | | | | | | | | |
| Anemia | 2(22.2) | 0 | 0 | 1(14.3) | 0 | 0 | 0 | 1(20) | 0 |  | 0 | 0 | 0 | 0 | 0 | 0 |
| Respiratory diseases | | | | | | | | | | | | | | | | |
| Anoxia | 0 | 0 | 0 | 0 | 0 | 0 | 0 | 0 | 0 |  | 0 | 0 | 1(20) | 0 | 0 | 0 |
| Metabolic and nutritional diseases | | | | | | | | | | | | | | | | |
| hypokalemia | 0 | 0 | 0 | 0 | 0 | 0 | 0 | 1(20) | 0 |  | 0 | 0 | 0 | 0 | 0 | 0 |
| Loss of appetite | 1(11.1) | 0 | 0 | 0 | 0 | 0 | 1(9.1) | 0 | 0 |  | 0 | 0 | 0 | 0 | 0 | 0 |
| Hyperuricemia | 0 | 0 | 0 | 0 | 0 | 0 | 0 | 0 | 0 |  | 0 | 0 | 0 | 1(3.1) | 0 | 0 |
| Nervous system disease | | | | | | | | | | | | | | | | |
| Dizzy | 1(11.1) | 0 | 0 | 0 | 0 | 0 | 1(9.1) | 0 | 0 |  | 0 | 1(20) | 0 | 1(3.1) | 0 | 0 |
| Neuralgia | 0 | 0 | 0 | 0 | 0 | 0 | 0 | 0 | 0 |  | 0 | 0 | 0 | 1(3.1) | 0 | 0 |
| Syncope | 0 | 0 | 0 | 0 | 0 | 0 | 0 | 0 | 0 |  | 0 | 0 | 1(20) | 0 | 0 | 0 |
| Musculoskeletal and connective tissue diseases | | | | | | | | | | | | | | | | |
| Backache | 0 | 0 | 0 | 0 | 1(50) | 0 | 3(27.3) | 0 | 0 |  | 0 | 0 | 0 | 4(12.5) | 0 | 0 |
| Ostealgia | 2(22.2) | 0 | 0 | 1(14.3) | 1(50) | 0 | 0 | 0 | 0 |  | 0 | 0 | 0 | 1(3.1) | 1(16.7) | 0 |
| Cervicodynia | 0 | 0 | 0 | 1(14.3) | 0 | 0 | 0 | 0 | 0 |  | 0 | 0 | 0 | 0 | 0 | 0 |
| Physical pain | 0 | 0 | 0 | 0 | 0 | 0 | 0 | 0 | 0 |  | 0 | 0 | 0 | 2(6.3) | 0 | 0 |
| Musculoskeletal pain | 0 | 0 | 0 | 0 | 0 | 0 | 1(9.1) | 0 | 0 |  | 0 | 0 | 0 | 0 | 0 | 0 |
| Systemic diseases and various reactions at the site of administration | | | | | | | | | | | | | | | | |
| Weak | 2(22.2) | 0 | 0 | 1(14.3) | 0 | 0 | 1(9.1) | 0 | 0 |  | 0 | 0 | 0 | 0 | 0 | 0 |
| Fever | 0 | 0 | 0 | 3(42.9) | 0 | 0 | 2(18.2) | 1(20) | 0 |  | 1(3.4) | 0 | 0 | 1(3.1) | 0 | 0 |
| Chest pain | 0 | 0 | 0 | 0 | 0 | 0 | 0 | 0 | 0 |  | 0 | 0 | 0 | 1(3.1) | 0 | 0 |
| Pain | 0 | 1(100) | 0 | 0 | 0 | 0 | 0 | 1(20) | 0 |  | 0 | 0 | 0 | 2(6.3) | 0 | 0 |
| Infectious and infectious diseases | | | | | | | | | | | | | | | | |
| Upper respiratory tract infection | 1(11.1) | 0 | 0 | 0 | 0 | 0 | 0 | 0 | 0 |  | 0 | 0 | 0 | 0 | 0 | 0 |
| Hepatobiliary disease | | | | | | | | | | | | | | | | |
| Abnormal liver function | 0 | 0 | 0 | 0 | 0 | 0 | 0 | 0 | 0 |  | 1(3.4) | 0 | 1(20) | 1(3.1) | 1(16.7) | 1(100) |
| Heart disease | | | | | | | | | | | | | | | | |
| External ventricular contraction | 0 | 0 | 0 | 0 | 0 | 0 | 0 | 0 | 0 |  | 1(3.4) | 0 | 0 | 0 | 0 | 0 |
| Immune system disease | | | | | | | | | | | | | | | | |
| Drug hypersensitivity | 0 | 0 | 0 | 0 | 0 | 0 | 1(9.1) | 0 | 0 |  | 0 | 0 | 0 | 0 | 0 | 0 |
